# Squamous Cell Carcinoma of the Ovary: A Rare Case

**DOI:** 10.7759/cureus.74547

**Published:** 2024-11-26

**Authors:** Chrysoula Margioula-Siarkou, Emmanouela-Aliki Almperi, Aristarchos Almperis, Georgia Margioula-Siarkou, Georgios Titilas, Konstantinos Dinas, Stamatios Petousis

**Affiliations:** 1 2nd Department of Obstetrics and Gynecology, Gynecologic Oncology Unit, Ippokrateio General Hospital of Thessaloniki, Aristotle University of Thessaloniki, Thessaloniki, GRC

**Keywords:** carcinoma, case report, dermoid cyst, ovary, squamous cell

## Abstract

Ovarian squamous cell carcinoma (SCC) is a rare entity among primary ovarian cancers. This type of cancer typically originates from the transformation of mature cystic teratomas, commonly known as dermoid cysts, and occasionally from associations with endometriosis or Brenner’s tumors. The typical clinical scenario involves presentation in postmenopausal women, with symptoms arising from tumor growth or metastasis. Herein, we present a case study of SCC arising from a dermoid cyst in the right ovary. Alongside this, we offer a concise review covering the histogenesis, diagnostic approaches, current therapeutic modalities, and prognosis associated with this condition. A 62-year-old woman presented with abdominal pain and fever. Imaging revealed a large mass originating from the right ovary, suspected to be ovarian serous cystadenocarcinoma. Elevated CA 19-9 levels indicated malignancy. The case was discussed in a multidisciplinary tumor board (MTB), leading to diagnostic laparoscopy. Despite initial biopsy results suggesting no malignancy, PET-CT indicated possible ovarian malignancy. Further exploration via exploratory laparotomy confirmed the malignancy through fast-track biopsy. As a result, intraoperatively, a primary debulking surgery was decided. The final diagnosis was primary moderately differentiated squamous ovarian carcinoma, stage IIB, originating from a dermoid cyst. The patient was referred for chemotherapy and is currently under follow-up care. This case underscores the complexity of ovarian cancer diagnosis and the importance of multidisciplinary approaches in treatment decisions. As of now, there are no established treatment guidelines for the effective management of this histotype. More research specifically tailored to this aim, involving global contribution and extended follow-up periods, are essential to establish the best management strategies.

## Introduction

According to the literature, squamous cell carcinoma (SCC) of the ovary represents an unusual condition, with a frequency of below 1% of all primary malignant ovarian tumors [1]. The majority concerns cases originating from the malignant transformation of mature cystic teratomas, dermoid cysts, and less frequently is associated with endometriosis or Brenner’s tumor [2,3]. Nevertheless, pure or primary ovarian SCC, which is not linked to any of the aforementioned preexisting ovarian conditions, has also been described [4]. The typical presentation refers to a postmenopausal woman, with an average age of 52.9 years, most commonly presenting for consultation due to symptoms associated with tumor growth or metastasis [5]. To our knowledge, no more than 42 cases of SCC have been described until now [6].

The clinical manifestation varies depending on the spread of the disease and the clinical stage. Early-stage cases may display nonspecific presentation such as diffuse pelvic or abdominal tenderness. In more advanced stages, abdominal distension may occur due to the presence of multiple tumoral masses, visceral compression, or invasion. Consequently, manifestations in patients at advanced stages may vary from palpable abdominal mass, vaginal bleeding, and dysfunction in the bowel, colon, or urinary bladder, while some symptoms are nonspecific, including weight loss and fever [7,8]. As far as the treatment of this entity is concerned, the infrequency of cases has led to the absence of a standardized therapeutic protocol. Experience in this domain remains limited to individual case reports or case series.

Herein, we report a case of SCC arising from a dermoid cyst of the right ovary, including a short review of histogenesis, diagnosis, current treatment options, and prognosis.

## Case presentation

**Figure 1:**
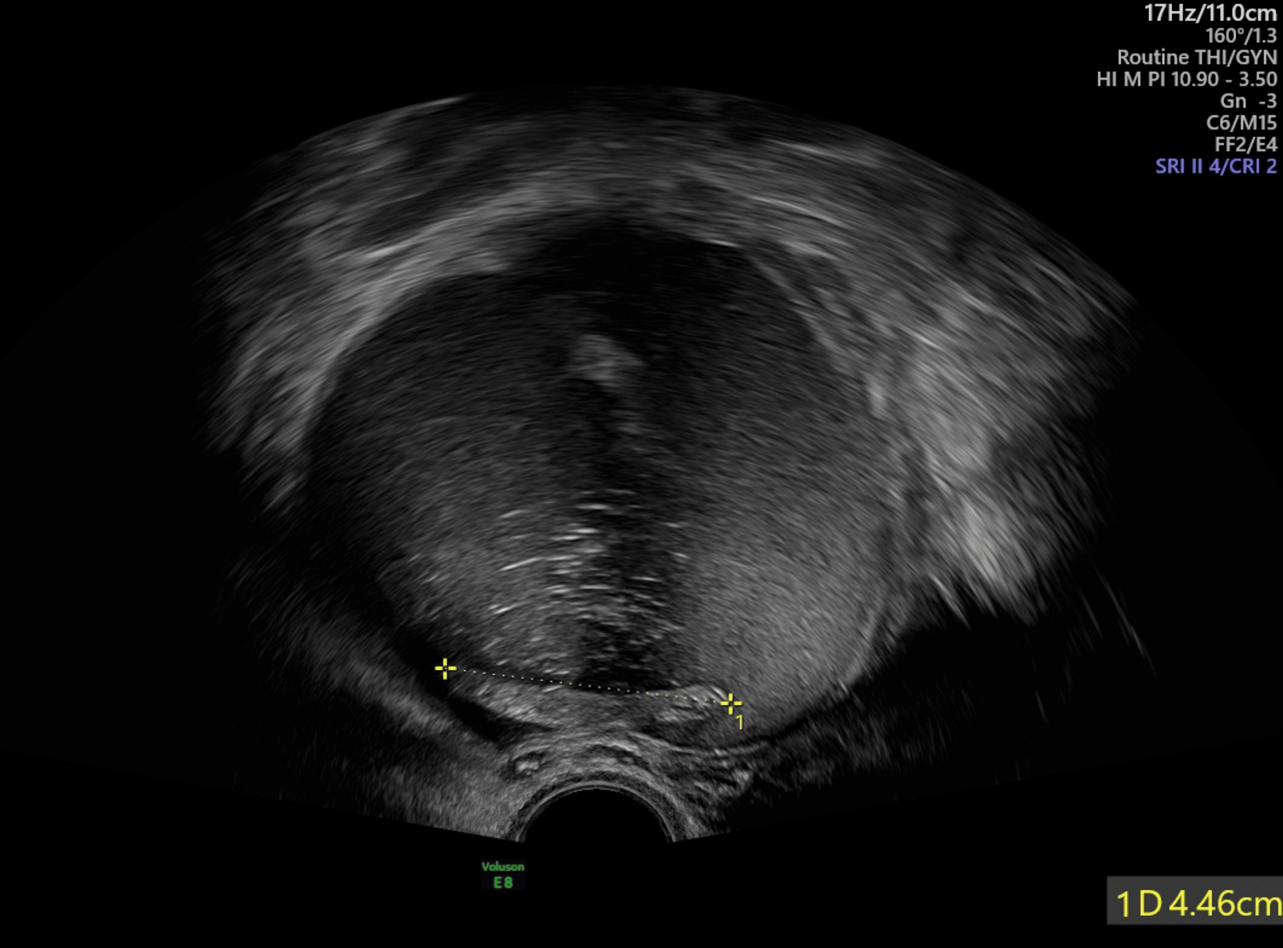
Abdominal ultrasound depicting the mass of 10 cm in diameter

A 62-year-old woman presented to our department with abdominal pain and fever asking for further consultation and management. Her personal history included allergic asthma, dyslipidemia, and appendectomy. During clinical examination, an abdominal mass originating from the right ovary was palpated, a finding that was revealed at first by abdominal ultrasound, described as a multicompartmental heterogeneous formation, with a diameter of 12 cm in the anatomical region of the right ovary. Ultrasonographic features suggested the possibility of malignancy (Figure 1).

Following a CT scan, the finding was verified and was most likely attributed to an ovarian serous cystadenocarcinoma, which compressed the right ureter. Regarding tumor markers, only CA 19-9 was extremely high, over 19790 U/mL, often seen in mucinous or borderline ovarian tumors. As the gynecologic oncology unit of our department represents a European Society of Gynaecological Oncology (ESGO)-accredited department for gynecologic oncology, all cases treated are discussed in our multidisciplinary tumor board (MTB). This case was also reviewed in our MTB with the copresence of gynecologic oncologists, radiation oncologists, medical oncologists, pathologists, and radiologists where a diagnostic laparoscopy was decided. The Fagotti score performed during surgery was 10/14 (the hemidiaphragm, peritoneum, omentum, small intestine, and mesentery), and biopsies from the peritoneum and omentum were obtained, while no sign of endometriosis or endometrioma was observed, excluding them from the differential diagnosis. The histopathological report excluded the incidence of malignancy, and as a consequence, a further investigation with PET-CT was performed, which revealed a possible ovarian malignancy with bulky pelvic lymph nodes and peritoneal carcinomatosis.

Taking the above into consideration, the patient was reviewed again in our MTB where an exploratory laparotomy was decided. During the surgery, a right salpingo-oophorectomy and drainage of a right tubo-ovarian abscess were performed, and the mass was sent for a fast-track biopsy, which confirmed the malignancy. As a result, intraoperatively, a primary debulking was decided, including a total hysterectomy with bilateral salpingo-oophorectomy, infragastric omentectomy, the excision of round ligament of the liver, the excision of trocar incision sites, the excision of a part of the parietal peritoneum and of an epiploic appendix, and finally the excision of the sigmoid colon and rectum resulting to an end colostomy. According to histopathology,** **squamous components were present, which can be seen even in serous, endometrioid, clear cell, or mucinous carcinomas. Therefore, the final answer was given by immunohistochemistry, which revealed positive p63, cytokeratin (CK) 5/6, and CK34bE12 markers. The diagnosis of a primary squamous ovarian carcinoma that was moderately differentiated, stage IIB according to the International Federation of Gynecology and Obstetrics (FIGO) classification, arose from a dermoid cyst, which expanded to retroperitoneal fat, and serosa of sigmoid was finally made. By examining postoperatively the CT scan, chest X-rays, and head and neck and under the absence of p16 marker by immunochemistry, the possibility of secondary or metastatic cervical cancer to the ovary was ruled out. Following the new MTB decision, the patient was subsequently referred for chemotherapy. The patient is being monitored without complications both surgically and oncologically. The current overall survival (OS) is three months.

Written informed consent was obtained from the individual(s) for the publication of any potentially identifiable images or data included in this article.

## Discussion

We presented a case of a woman who presented to our department for consultation due to epigastric pain and fever. Following a diagnostic laparoscopy and a PET scan, she underwent a debulking procedure to achieve zero residual mass and received chemotherapy, since she was diagnosed with ovarian SCC originating from a dermoid cyst, teratoma. Ovarian carcinomas rank as the second most common carcinoma in the female reproductive tract [1]. Primary ovarian carcinomas encompass various histological types, with high-grade serous being the most common (68%-71%), followed by clear cell (12%-13%), endometrioid (9%-11%), mixed (6%), low-grade serous (3%-4%), mucinous (3%), transitional (1%), and SCC, which represents less than 1% [1,8].

In most cases, primary SCC of the ovary arises from dermoid cysts. While mature teratomas constitute 20% of ovarian tumors, their malignant transformation occurs in no more than 5.5% of cases, with up to 75% of these transformations resulting in squamous cell carcinomas [9]. The malignant transformation of ovarian teratoma can originate from any germ cell type within these tumors. Consequently, any of the following histopathological types may manifest: adenocarcinomas, squamous cell carcinomas, sarcomas, melanomas, adenosquamous carcinomas, or carcinoid tumors. Nevertheless, it is noteworthy that the vast majority of cases (up to 80%) display invasive characteristics, such as increased size and radiological signs of intra-tumoral vascularization, which are observed to be SCC [10,11]. Furthermore, it has been noted that SCC arising from endometriosis predominantly originates from foci in the ovary and vaginal septum.

The literature consistently emphasizes its aggressive nature, often associated with a low survival rate of less than 20% within six months [5]. In addition, several authors have proposed high-risk human papillomavirus (HPV) infection as the prevalent oncogenic factor associated with the development of ovarian SCC, since between 36% and 52% of patients are HPV-positive [8]. Few reports exist on high-risk HPV-related ovarian squamous cell carcinoma, mostly individual case studies, suggesting a potential link between HPV and ovarian SCC. Some studies, such as Pins et al. [12], found HPV DNA in reproductive tract tissues, including ovarian tumors, while others reported synchronous lesions in the cervix and ovaries [13,14]. Additionally, there are also studies reporting histogenesis of ovarian SCC either from spread through the transcoelomic route of cervical intraepithelial lesions along the mucosal surface of the female genital tract [15] or the possibility of micrometastasized disease to the ovary, originating from microinvasive and angioinvasive cervical SCC [16].

The differential diagnosis encompasses primary ovarian lesions with squamous differentiation. Among them are endometrioid carcinoma with squamous differentiation, squamous cell carcinoma associated with mature cystic teratoma, ovarian carcinosarcoma (malignant mixed mesodermal tumor), endometriosis, and metastasis from another organ or anatomical location such as the cervix. All of them were ruled out in our case, with the help of imaging modalities and histopathological and finally immunochemistry assays. According to the literature, recent advancements regarding the molecular profile of this type of tumor may help us make a diagnosis and personalize the treatment. One of the critical mutations driving this malignancy is found in the *TP53* gene, which disrupts cellular mechanisms for DNA repair and apoptosis regulation, playing a significant role in tumor development [17]. Furthermore, epidermal growth factor receptor (EGFR) overexpression or mutations are commonly observed, contributing to unchecked tumor growth through dysregulated activation of the epidermal growth factor receptor pathway [17]. The protein p63, a p53 family member, is also often expressed, aiding in squamous epithelial differentiation and serving as a diagnostic marker for squamous cell origin [18]. Additionally, mutations in the *PIK3CA* gene, which activate the phosphatidylinositol 3-kinase/protein kinase B (PI3K/AKT) signaling pathway, have been reported, further driving cell proliferation and survival [19]. Together, these molecular changes highlight the aggressive nature of primary ovarian SCC and assist in differentiating it from other ovarian cancers.

Distinguishing primary from secondary SCC may pose a more intricate challenge. Primary SCC of the ovary typically arises from malignant transformation within a mature cystic teratoma and is characterized by molecular alterations such as *TP53* and EGFR mutations, with no significant association with HPV, unlike SCCs in other anatomical locations [17]. In contrast, secondary SCC of the ovary, which results from metastasis of HPV-driven malignancies such as cervical SCC, retains the HPV-related genetic signatures, including the involvement of E6/E7 oncoproteins that inactivate p53 and retinoblastoma protein (RB) [20]. Secondary SCC may also arise from non-squamous ovarian tumors such as mucinous neoplasms or endometriosis, exhibiting mutations such as *KRAS* and *PTEN*, with subsequent squamous differentiation [21]. Hence, while primary ovarian SCC shows squamous-specific mutations independent of HPV, secondary SCC molecular profiles are highly reflective of their primary tumor origin, including HPV-driven pathways or preexisting ovarian pathologies.

Excluding a metastatic involvement requires performing a thorough clinical examination and imaging of the upper gastrointestinal tract, thoracic cavity, head and neck, bladder, and skin. In our case, the immunohistochemistry was compatible with an SCC, while in the PET scan, no other implants were observed but the peritoneal carcinomatosis, while a careful examination of the imaging results accompanied by the immunochemistry ruled out occult primary sites of tumor. Furthermore, some authors have explored the sensitivity and specificity of squamous cell carcinoma antigen as a potential marker for detecting potentially at-risk cases. Nevertheless, this benchmark is still a moot point, and the establishment of a definitive cutoff value has not been achieved yet [22,23]. The malignant transformation of mature cystic teratomas tends to produce larger tumors and affects younger patients compared to benign forms [20]. Diagnosis typically occurs postoperatively through pathological examination, as clinical and radiological methods are insufficient for preoperative identification. CT and MRI are commonly used to diagnose mature cystic teratomas before surgery, but distinguishing between benign and malignant forms is challenging, although useful information on disease extent is provided [7].

Up to date, there are currently no established treatment guidelines for the effective management of pure squamous cell carcinoma (SCC) of the ovary due to the rarity of this tumor. A multimodality approach, including aggressive cytoreductive surgery, followed by cisplatin-based chemotherapy and/or radiotherapy, has been suggested as a potential strategy to enhance survival [8]. In over 75% of cases, the treatment for SCC typically involves total hysterectomy and bilateral adnexectomy, with or without omentectomy. When considering the surgical approach, it seems that the open approach is preferable. Although the minimally invasive route may be attractive for its potential benefits in patient recovery, some authors have stressed the importance of avoiding this procedure. Unless there is a confident and well-trained surgeon, the probability of spillage and cystic rapture during laparoscopic handling or the extraction of the specimen from the abdomen is high [24,25]. In cases where the laparoscopic approach is chosen and spillage occurs, thorough peritoneal washing is recommended to reduce the risk of additional peritoneal lesions. Recent studies suggest that minimally invasive surgery is safe if the specimen is securely placed in an endoscopic retrieval bag [9]. Regarding the extent of the operation, obtaining peritoneal biopsies to achieve accurate staging is widely accepted by many authors, while lymphadenectomy lacks acceptance due to the non-preferential involvement of the lymphatic route in this type of tumor [26,27]. On the other hand, retrospective data from a large systematic review and meta-analysis demonstrated a potential survival advantage from lymph node dissection (mean survival of “with lymphadenectomy” group versus “without”: 59.2 versus 40.4 months) [2]. For patients with stage IB tumors and beyond, a combination of platinum-based chemotherapy with paclitaxel or gemcitabine may enhance survival. Considering the radiosensitivity of squamous tumors, pelvic radiotherapy could be considered as an additional treatment. The efficacy of optimal debulking surgery to overall survival is well-established.

Given the advancement in molecular analysis, targeted therapies are added arrows in our quiver. Poly adenosine diphosphate (ADP)-ribose polymerase (PARP) inhibitors, such as olaparib and niraparib, are well-established to target DNA repair deficiencies in tumors with homologous recombination repair defects, particularly those involving mutations in *BRCA1* and *BRCA2* in ovarian carcinoma; its use may be beneficial given the similar DNA repair deficiencies in rare histological subtypes, including SCC [28]. Additionally, alterations in *PIK3CA* activate the PI3K/AKT/mammalian target of rapamycin (mTOR) signaling pathway, making PI3K inhibitors (e.g., alpelisib) a viable therapeutic approach [29,30]. Novel therapies, such as immune checkpoint inhibitors, for example, pembrolizumab, are also being investigated for tumors with a high mutational load, focusing on programmed cell death protein 1 (PD-1)/programmed death-ligand 1 (PD-L1) pathways. Research is ongoing to further define the molecular characteristics of ovarian SCC, paving the way for more personalized treatment strategies [31,32].

Predicting long-term outcomes in such rare cases, however, depends heavily on the progression of the disease at the time of diagnosis, the potential intraperitoneal leakage and consequently the spread of the tumor, the vascular invasion, the surgical staging postoperatively, the potential residual mass, and the pattern of growth [7,10]. Still, despite the abovementioned interventions, the five-year survival rate at stages II and beyond is no more than 34% [8].

Future research on ovarian squamous cell carcinoma (SCC) should focus on clinical trials and targeted therapies due to its aggressive nature. Trials could explore personalized treatments, including molecularly targeted therapies for genetic mutations involved in SCC. Investigating the potential link between human papillomavirus (HPV) and ovarian SCC is also crucial, as HPV’s role in other SCCs suggests it may contribute here.

Given the absence of structured guidelines, with our presentation, it is strongly proposed that for rare cases with uncertain diagnosis and high possibility of malignancy, a thorough clinical examination helped by imaging assessment and compliance with the principles of oncology should be strictly followed. This is why such cases are highly recommended to be treated in gynecologic oncology units, where MTB is held, such as our department. Histopathology and immunochemistry are essential for the final diagnosis, which may be made postoperatively. The importance of an experienced surgeon is undeniable since proper surgical staging and optimal debulking, leaving no residual disease behind, are crucial for the survival of the patient. Complying with these principles and being vigilant are key points for effective management.

## Conclusions

This case report of primary SCC of the ovary highlights the diagnostic and therapeutic challenges associated with this rare and aggressive malignancy. It aims to raise awareness among gynecologists due to the rarity and unfavorable prognosis associated with ovarian SCC, which is seen especially in postmenopausal women. Careful diagnostic workup and discussion in an MTB to plan a therapeutic strategy are essential steps until histopathological and immunochemistry reports establish the diagnosis. Complying with the principles of oncologic surgery for optimal cytoreduction is deemed essential at all stages, while implementing adjuvant chemotherapy in stage II-IV disease seems to have a pivotal role in improving survival. Adding adjuvant radiotherapy or chemotherapy lacks clear establishment, with the second showing variability among the few published progressed cases.
